# Pretreatment CT Texture Parameters as Predictive Biomarkers of Progression-Free Survival in Follicular Lymphoma Treated with Immunochemotherapy and Rituximab Maintenance

**DOI:** 10.3390/diagnostics13132237

**Published:** 2023-06-30

**Authors:** Carole Durot, Eric Durot, Sébastien Mulé, David Morland, François Godard, Anne Quinquenel, Alain Delmer, Philippe Soyer, Christine Hoeffel

**Affiliations:** 1 Department of Radiology, Reims University Hospital, 45 Rue Cognacq-Jay, 51092 Reims, France; 2 Department of Hematology, Reims University Hospital, 45 Rue Cognacq-Jay, 51092 Reims, France; 3 Department of Radiology, Henri Mondor University Hospital, 51 Avenue du Maréchal de Lattre de Tassigny, 94010 Créteil, France; 4 Faculté de Médecine, Université Paris-Est Créteil, 61 Avenue du Général de Gaulle, 94000 Créteil, France; 5 Department of Nuclear Medicine, Godinot Institute, 1 Rue du Général Koenig, 51100 Reims, France; 6 CReSTIC, EA 3804, University of Reims Champagne-Ardenne, UFR Moulin de la Housse, 51867 Reims, France; 7 Department of Radiology, Hôpital Cochin, AP-HP, 27 Rue du Faubourg Saint-Jacques, 75014 Paris, France; 8 Faculté de Médecine, Université Paris Cité, 75006 Paris, France

**Keywords:** follicular lymphoma, positron emission tomography, tomography, X-ray computed

## Abstract

The purpose of this study was to determine whether texture analysis features present on pretreatment unenhanced computed tomography (CT) images, derived from 18F-fluorodeoxyglucose positron emission/computed tomography (18-FDG PET/CT), can predict progression-free survival (PFS), progression-free survival at 24 months (PFS 24), time to next treatment (TTNT), and overall survival in patients with high-tumor-burden follicular lymphoma treated with immunochemotherapy and rituximab maintenance. Seventy-two patients with follicular lymphoma were retrospectively included. Texture analysis was performed on unenhanced CT images extracted from 18-FDG PET/CT examinations that were obtained within one month before treatment. Skewness at a fine texture scale (SSF = 2) was an independent predictor of PFS (hazard ratio = 3.72 (95% CI: 1.15, 12.11), *p* = 0.028), PFS 24 (hazard ratio = 13.38; 95% CI: 1.29, 138.13; *p* = 0.029), and TTNT (hazard ratio = 5.11; 95% CI: 1.18, 22.13; *p* = 0.029). Skewness values above −0.015 at SSF = 2 were significantly associated with lower PFS, PFS 24, and TTNT. Kurtosis without filtration was an independent predictor of PFS (SSF = 0; HR = 1.22 (95% CI: 1.04, 1.44), *p* = 0.013), and TTNT (SSF = 0; hazard ratio = 1.23; 95% CI: 1.04, 1.46; *p* = 0.013). This study shows that pretreatment unenhanced CT texture analysis-derived tumor skewness and kurtosis may be used as predictive biomarkers of PFS and TTNT in patients with high-tumor-burden follicular lymphoma treated with immunochemotherapy and rituximab maintenance.

## 1. Introduction

Follicular lymphoma (FL) is the second most common subtype of non-Hodgkin lymphoma, representing approximatively 20 to 30% of lymphomas [1]. FL mainly spreads to lymph nodes, but the spleen, bone marrow, peripheric blood, or Waldeyer ring can also be involved. The diagnosis of FL is made using histopathological analysis of a lymphadenopathy or any other involved organ [2]. According to the World Health Organization classification, FLs are classified into grades 1–2 and 3, according to the number of centroblasts, with a further histological subdivision into grades 3a and 3b [3]. While grade 3b FLs are rare and treated similarly to aggressive diffuse large B-cell lymphomas, grade 1–2 and 3a FLs are considered indolent and incurable. Immunochemotherapy followed by rituximab maintenance is widely accepted as a standard of care for the latter cases [4,5,6,7,8]. A baseline 18F-fluorodeoxyglucose positron emission tomography/computed tomography examination (18F-FDG PET/CT) is performed upon diagnosis to determine the extent of the disease and the metabolic burden [9].

A treatment approach combining chemotherapy and rituximab has resulted in an improved 5-year survival rate of up of 80 to 90% and in a median survival time of 10 to 12 years in patients with FL [10,11,12]. However, approximately 20% of patients treated with immunochemotherapy still have a more aggressive course, with early progression (i.e., occurring within two years) and a 5-year overall survival (OS) of only 50% [13]. Identification of these patients is currently the main therapeutic issue, as they could benefit from either early intensified treatment or a shift to new therapeutic agents [13,14].

Current prognostic biomarkers, including the Follicular Lymphoma International Prognostic Index (FLIPI) [15], FLIPI2 [16] and the PRIMA-Prognostic Index (PRIMA-PI) are limited in their identification of patients at high risk of early progression. Recently, baseline total metabolic tumor volume (T-MTV) values above the threshold of 510 cm^3^ on 18F-FDG PET/CT, combined with FLIPI2, have demonstrated potential for identifying these patients [17]. However, T-MTV measurement is time-consuming and not performed in routine practice [17,18,19,20]. There is thus still a need for the identification of baseline predictive biomarkers, particularly of progression-free survival (PFS) and progression-free survival at 24 months (PFS24).

CT texture analysis (TA) is a relatively recently emerging technique for quantifying tumor heterogeneity based on an analysis of the distribution and relationship of pixel gray levels in the image [21,22]. CT TA can provide information regarding survival and response to treatment for many solid cancer types, such as colorectal [23], melanoma [24,25], esophageal [26], head and neck [27], non-small-cell lung [28,29], cerebral [30], or hepatocellular carcinoma [31,32]. However, data regarding hematological malignancies are scarce. Only one study, dealing with all types of lymphomas, has shown a promising role for CT TA from interim 18F-FDG-PET/CT data, in indicating kurtosis as a predictive biomarker of relapse, but the study included only a limited number of patients with various types of lymphomas [33]. To date, the role of CT TA has never been specifically investigated in a population of patients with FL.

The goal of this study was thus to assess whether TA parameters obtained from CT images during 18F-FDG PET/CT examination before treatment initiation are independently associated with survival in patients with high-tumor-burden FL treated with immunochemotherapy and rituximab maintenance.

## 2. Materials and Methods

### 2.1. Study Population

This study was performed in accordance with the Declaration of Helsinki of the World Medical Association revised in 2013 for experiments involving humans and declared to the “Commission Nationale Informatique et Libertés” (authorization number 1118523; 9 March 2020). Participants were systematically informed that their data were being collected for an anonymous retrospective study and that they could refuse to participate in the study (i.e., non-opposition statement).

All consecutive patients with high-tumor-burden FL treated between July 2010 and September 2016 were retrieved from our institutional database. Patients were included in this study when they met the following criteria: (i) patient had a grade 1–2 or 3a FL confirmed on biopsy, with a high tumor burden according to GELF criteria (Groupe d’Etude des Lymphomes Folliculaires); (ii) patient was treated using immunochemotherapy for induction, followed by rituximab as maintenance therapy; and (iii) patient had undergone baseline 18F-FDG PET/CT within one month prior to treatment. This initial search retrieved a total of 88 patients. Patients were further excluded when (i) they had no accurately delineable lesions on baseline CT or (ii) when no 18F-FDG PET/CT examination performed less than one month before treatment was available for review. In total, 72 patients were ultimately included in the study.

For all patients, clinical, biological, and imaging data were recorded. They included the patient’s age, gender, Eastern Cooperative Oncology Group (ECOG) performance status, B symptoms, serum level of B2-microglobuline and platelets, presence of circulating tumor cells, the longest diameter of the largest involved node (LoDLIN), presence of extra-nodal involvement, FLIPI and FLIPI2 scores with FLIPI risk category (FLIPI RC), maximum standardized uptake value (SUVmax), and T-MTV on baseline 18F-FDG PET/CT images.

### 2.2. Follow-Up and Endpoints

All patients underwent clinical, biological, and radiological follow-up according to our local policy. The primary endpoint (PFS) was defined as the time from initiation of immunochemotherapy to clinical, biological, or imaging progression, or death from any cause. The secondary endpoint (PFS24) was defined as being alive and progression-free 24 months after initiation of the treatment. Time to next treatment (TTNT), which was defined as the time from initiation of the treatment to the initiation of the second-line treatment in case of progression, and OS, which was defined as the time from initiation of the treatment to death, were also used as evaluation criteria.

For PFS, patients without recurrence at the end of the follow-up period were censored at that time. For PFS 24, patients with no progression at 24 months were censored at that time. For OS, patients who were alive at the end of follow-up were censored.

### 2.3. 18F-FDG PET/CT Acquisition and Analysis

All patients underwent a skull-base-to-proximal-thigh 18F-FDG PET/CT examination, 60 min after intravenous administration of 5 MBq/kg of 18F-FDG. 18F-FDG PET/CT examinations were performed using a Philips Gemini Dual system (Philips Healthcare, Eindhoven, the Netherlands). CT data of PET/CT examinations (120–140 kV, 100–150 mAs) were reconstructed using filtered back projection (FBP) and a medium smooth filter kernel (B30) on a 512 × 512 matrix (voxel size in axial plane, 1.17 × 1.17; thickness, 6.5 mm). 18F-FDG PET/CT examinations were retrospectively reviewed by two nuclear medicine physicians (F.G. and D.M.) with, respectively, four and nine years of experience in nuclear medicine, blinded to patient outcome on a dedicated interpretation console (AW Server, General Electric Healthcare, Milwaukee, WI, USA) with PET/CT images scaled to a fixed standard uptake value (SUV). For each patient, up to five target lesions (lymph nodes or spleen) were selected to perform TA based on their high uptake values. The highest SUVmax among all lesions was recorded. T-MTV was computed through aggregating the metabolic volumes of all local nodal and extranodal lesions according to Meignan et al. [17]. A 41% SUVmax threshold was used. Discrepancies were resolved using consensus opinion of the two physicians.

### 2.4. CT Texture Analysis

**Figure 1:**
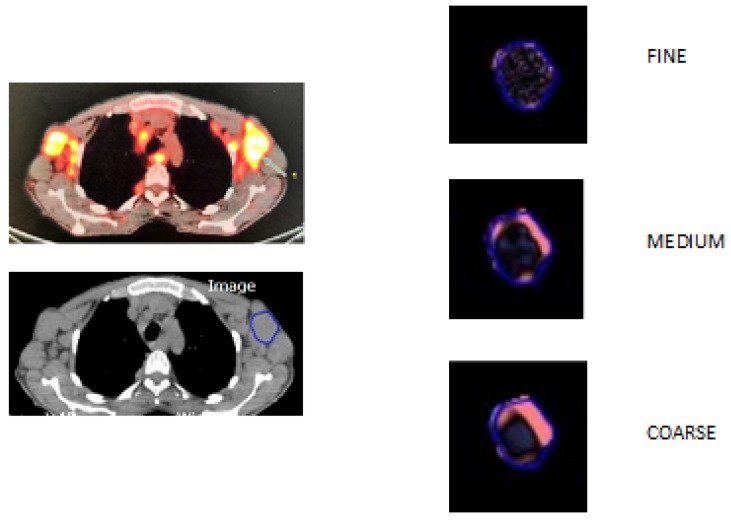
Illustration of lesion delineation, image filtration at fine, medium, and coarse texture scales.

CT TA was made on pretreatment unenhanced CT images extracted from baseline 18F-FDG PET/CT examinations using a commercially available version of TexRAD software (TexRAD Ltd., London, UK). A radiologist (C.D.) with six years of experience in oncological imaging selected the image displaying the largest cross-sectional area and placed a region of interest (ROI) encompassing entirely each target lesion (Figure 1). One to five target lesions were analyzed for each patient.

CT TA was made using a two-step procedure. First, a Laplacian-of-Gaussian spatial band-pass filter was applied in order to allow extraction of features at fine (SSF = 2, object radius of 2 mm), medium (SSF = 3–5, object radius of 3–5 mm), and coarse (SSF = 6, object radius of 6 mm) scales. Then, five parameters were obtained from the quantification of the histogram distribution within the ROI. They included mean gray-level intensity (mean), standard deviation (SD), entropy, kurtosis, and skewness. For each patient, the mean value of each texture parameter among the selected lesions was calculated.

### 2.5. Statistical Analysis

Statistical analyses were performed using R software (version 3.4.1; R Development Core Team, R Foundation for Statistical Computing, Vienna, Austria). Quantitative variables were expressed as medians, interquartile ranges (Q1, Q3), and ranges. Categorical variables were expressed as raw numbers, proportions, and percentages [34].

Multivariable analysis was performed to identify independent variables associated with PFS, PFS24, TTNT, and OS among clinical, 18F-FDG PET/CT (T-MTV and SUVmax), and TA parameters. First, a multivariable L1 (least absolute shrinkage and selection operator-LASSO)-penalized Cox regression model was built to take into account the correlation between the estimates of each texture parameter from the different filter values and the small number of events compared with the number of included covariables [31]. The regularization parameter was determined using a 10-fold cross-validation. For variables non-related to outcome, the LASSO method shrunk down coefficient weights (CW) to zero coefficients. Variables with non-zero coefficients were potential predictors of outcome.

Finally, a multivariable Cox regression analysis was performed through integrating variables with non-zero coefficients selected via the LASSO model and clinico-biological variables as covariables, in order to estimate associated hazard ratios (HR) and their 95% confidence intervals (CI).

Kaplan–Meier analyses were also performed for each texture parameter predictor of outcome to identify an optimal threshold to discriminate between patients with good prognosis and those with a poor prognosis, using non-parametric log-rank tests. For all tests, *p*-values <0.05 were considered to indicate statistically significant differences.

## 3. Results

### 3.1. Patient Characteristics

Eighty-eight patients were eligible for the study. Sixteen patients who did not have a 18F-FDG PET/CT examination within one month prior to treatment were excluded. The final population included 72 patients (43 men and 29 women) with a median age of 61 years.

The main clinical, biological, nuclear medicine, and radiological characteristics of patients are reported in Table 1. The FLIPI, FLIPI2, and PRIMA-PI scores were, respectively, low for 10 (14%), 2 (6%), and 11 (22%) patients; intermediate for 28 (39%), 20 (57%), and 13 (27%) patients; and high for 34 (47%), 13 (35%), and 25 (51%) patients. FLIPI2 and PRIMA-PI were not available for all patients due to missing data (mainly information on bone marrow involvement).

The median T-MTV on baseline 18F-FDG PET/CT was 381 cm^3^ (range: 12–3329 cm^3^ Q1–Q3: 155–807 cm^3^), and 29 patients (40%) had a T-MTVT over the threshold of 510 cm^3^.

The median PFS and TTNT for the whole cohort were 7.1 years (95% CI: 3.8, not reached) and 7.5 years (95% CI: 6.8, 9.1), respectively. Thirteen patients (18%) progressed during the first 24 months after the initiation of the treatment. Five-year OS was 87%. Death occurred in 10 patients (14%), due to disease progression in 7 patients or to other causes not related to FL in 3 patients. Target lesions included lymphadenopathies, up to five, for every patient, and spleen in five patients.

### 3.2. Progression-Free Survival Analysis

At LASSO-penalized Cox regression analysis, two texture parameters were identified as potential predictors of PFS: kurtosis without filtration (CW, 0.03) and skewness at a fine scale (SSF = 2) (CW, 0.12), as well as two clinical parameters: patient sex (CW, 0.67) and ECOG performance status above 1 (CW, 0.98). Other features with non-zero coefficient weights included FLIPI category risk (CW, 0.21) and T-MTVT > 510 cm^3^ (CW, 0.21).

Multivariable Cox regression analysis confirmed kurtosis without filtration (SSF = 0; hazard ratio [HR], 1.22; 95% CI: 1.04, 1.44; *p* = 0.013), skewness at a fine texture scale (SSF = 2; HR, 3.72; 95% CI: 1.15, 12.11); *p* = 0.028), patient sex (HR, 4.81 (95% CI: 1.84, 12.55), *p* = 0.001) and ECOG performance status above 1 (HR, 5.53 (95% CI: 1.55, 19.79); *p* = 0.008) as independent predictors of PFS (Table 2).

**Figure 2:**
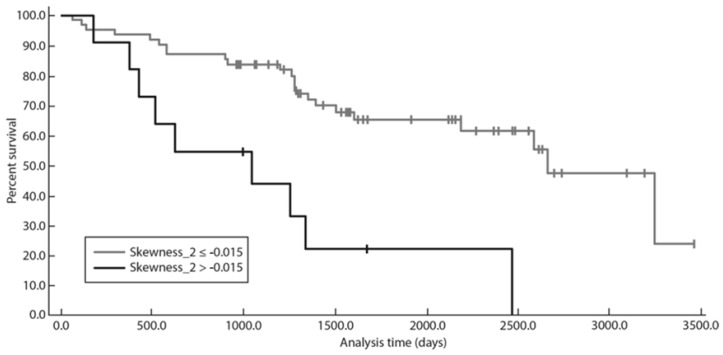
Graph shows Kaplan–Meier survival analysis for PFS with skewness at SSF = 2.

When dichotomized at the optimal threshold identified using Kaplan–Meier analysis, skewness above −0.015 at a fine texture scale (SSF = 2; *p* = 0.0016) was significantly associated with lower survival time (Figure 2).

### 3.3. Progression-Free Survival at 24 Months Analysis

LASSO-penalized Cox regression analysis identified three texture parameters as potential predictors of PFS24: kurtosis without filtration (SSF = 0) (CW, 0.09) and skewness at fine (SSF = 2) (CW, 0.69) and medium (SSF = 3) (CW, 0.65) texture scales. Two clinical parameters also highlighted non-zero coefficient weights: patient sex (CW, 0.42) and ECOG performance status above 1 (CW, 0.97), as well as FLIPI risk category (CW, 0.31) and LodLIN > 6 cm (CW, 0.32).

Multivariable Cox regression analysis confirmed skewness at a fine texture scale (SSF = 2; HR, 13.38; 95% CI: 1.29, 138.13; *p* = 0.029) as an independent predictor of PFS 24 (Table 3).

**Figure 3:**
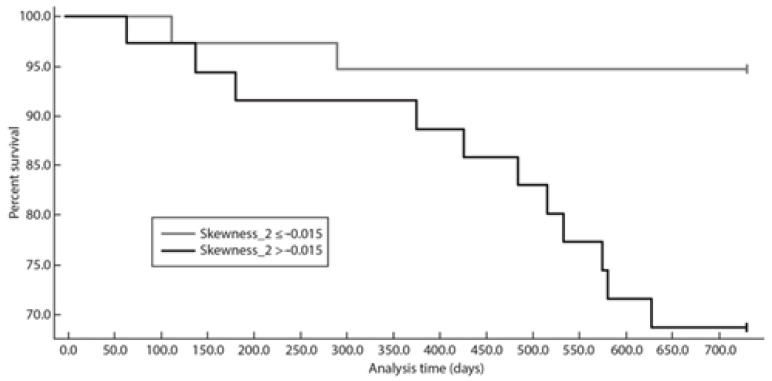
Graph shows Kaplan–Meier survival analysis for PFS24 with skewness at SSF = 2.

When dichotomized using the optimal threshold identified using Kaplan–Meier analysis, skewness > −0.015 at a fine texture scale (SSF = 2; *p* = 0.0086) was significantly associated with lower survival time (Figure 3).

### 3.4. Time to Next Treatment Analysis

At LASSO-penalized Cox regression analysis, kurtosis without filtration (CW, 0.04) and skewness at a fine scale (SSF = 2) (CW, 0.51) were identified as potential predictors of TTNT. Two clinical parameters also highlighted non-zero coefficient weights: patient sex (CW, 0.49) and ECOG performance status above 1 (CW, 1.16), as well as T-MTVT > 510 cm^3^ (CW, 0.35).

Multivariable Cox regression analysis then confirmed kurtosis without filtration (SSF = 0; HR, 1.23; 95% CI: 1.04, 1.46; *p* = 0.013), skewness at a fine texture scale (SSF = 2; HR, 5.11; 95% CI: 1.18, 22.13; *p* = 0.029), patient sex (HR, 3.72; 95% CI: 1.45, 9.53; *p* = 0.006), and ECOG performance status above 1 (HR, 5.90; 95% CI: 1.58, 21.98; *p* = 0.008) as independent predictors of TTNT (Table 4).

**Figure 4:**
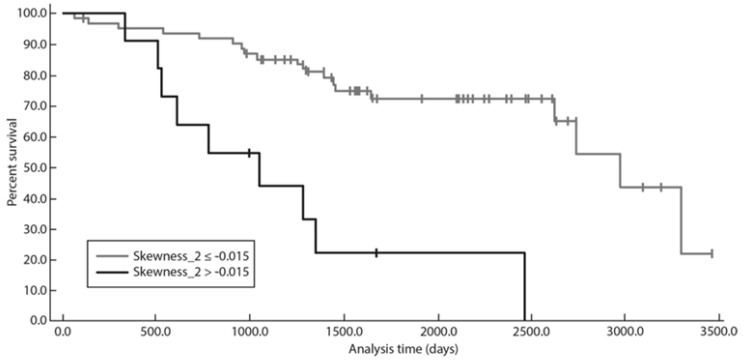
Graph shows Kaplan–Meier survival analysis for TTNT with skewness at SSF = 2.

When dichotomized using the optimal threshold identified at Kaplan–Meier analysis, skewness > −0.015 at a fine texture scale (SSF = 2; *p* = 0.0005) was significantly associated with lower survival time (Figure 4). Patients with skewness > −0.015 at SSF = 2 showed significantly poorer PFS, PFS 24, and TTNT (Figure 2, Figure 3 and Figure 4).

### 3.5. Overall Survival Analysis

LASSO penalized Cox regression analysis identified mean at a fine texture scale (SSF = 2) (CW, −3.85) as a potential predictor of OS. Three clinical parameters also highlighted non-zero CW: patient sex (CW, 5.25), ECOG performance status above 1 (CW, 2.93), and serum level of B2-microglobuline (CW, 8.18), as well as FLIPI category risk (CW, 4.17).

Multivariable Cox regression analysis confirmed patient sex (HR, 19.00; 95% CI: 1.66, 217.82; *p* = 0.018) and FLIPI category risk (HR, 6.43; 95% CI: 1.35, 30.66; *p* = 0.019) as independent predictors of OS (Table 5).

## 4. Discussion

Our study suggests that TA features of lymph nodes involved in high-tumor-burden FL extracted from baseline 18F-FDG PET/CT data are independent variables associated with PFS, PFS 24, and TTNT and may be used as predictors of outcome. Moreover, our study identified a single cut-off value of skewness that allows differentiating between patients with short survival time and those with long survival time in terms of PFS, PFS 24, and TTNT.

18F-FDG PET/CT is the reference imaging technique for baseline evaluation of patients with FL, mainly owing to the metabolic information that this technique provides. However, the quantitative information that may be extracted from associated CT data has not been exploited so far in the field of FL. Yet, CT TA is now considered as a new technique that allows a quantitative assessment of tumor heterogeneity, and many studies have recently shown its ability to predict PFS and OS in several types of cancer, as well as response to treatment [23,24,25,26,27,28,29,30,31,32,35].

Recent improvements in the treatment of FL have led to marked improvement in overall survival rate of patients affected by this condition [10,11,12]. The actual critical issue regarding FL involves a subset of approximately 20% of patients who have a more aggressive course of the disease under treatment and undergo disease progression within the first two years. As a consequence, there is active ongoing research for identifying baseline biomarkers that may help predict early relapse of high-tumor-burden FL. Several clinical prognostic indices have been developed during the last 20 years, but they are still limited to the prediction of early relapses. Baseline T-MTV, in combination with FLIPI2, has been reported as a strong predictor of outcome in patients with FL by Meignan et al. [17]. These researchers have even reported a cutoff of 510 cm^3^ that may discriminate between patients with good survival parameters and those with less favorable survival parameters. However, these results need to be further validated and their application in daily practice may be limited since T-MTV calculation is time-consuming and hardly feasible in routine [18,19].

Our analysis identified independent predictive value of skewness at a fine texture scale (SSF = 2) derived from target lesions of FL on unenhanced CT images for PFS, PFS 24, and TTNT. Skewness is an indicator of the asymmetry of the histogram corresponding to gray-level values within a specific ROI. A predominantly bright texture indicates a positive skewness, whereas predominantly dark texture indicates negative skewness [36,37]. In our study, greater values of skewness were associated with worse outcomes. Of note, we could identify a skewness threshold value above which patients had significantly poorer PFS, PFS 24, and TTNT. Our results parallel those obtained in other cancers for which high skewness at TA is a predictor of poorer PFS and OS [27,28,30,32,38].

The results of our current study are in line with those of prior studies as they confirm the independent predictive value of pretreatment kurtosis without filtration for PFS and TTNT in patients with high-tumor-burden FL [27,28,30,32,38]. Kurtosis reflects the peakedness/flatness of the histogram corresponding to the gray-level values within a specific ROI [37]. In our study, higher kurtosis values were associated with worse PFS and TTNT, in line with the results of the study by Ganeshan et al. [33]. In a series of patients with Hodgkin’s lymphoma and aggressive non-Hodgkin’s lymphoma, Ganeshan et al. showed that higher pretreatment kurtosis values measured on unenhanced CT images at a medium texture scale were associated with lower recurrence-free survival [33]. However, these researchers performed their study on both Hodgkin’s and aggressive non-Hodgkin’s lymphomas that do not share the same prognosis nor the same treatments as FLs in our population of patients. However, in line with other studies, our results provide additional evidence on the association between high kurtosis and poorer outcome in patients with FL [30,32,38,39].

In our study, patient sex, ECOG performance status > 1, and FLIPI category risk appeared as independent predictors of PFS, TTNT, and OS. Our results are in accordance with those of previous studies [16]. The predictive values of FLIPI2 and PRIMA-PI were not possible to assess in our study due to too much missing data. T-MTV above the threshold of 510 cm^3^ was not found to be an independent predictor of survival in our study, but the accuracy of this threshold remains debated for now.

Our study has some limitations. First, it is a retrospective, single-center study, with a limited number of patients. However, our cohort was homogeneous in terms of treatment, and complete metabolic analysis of 18F-FDG PET/CT was performed for each patient. A second limitation relates to TA made by a single observer only, thus possibly introducing bias due to the absence of interobserver variability assessment. A third limitation is the absence of a validation cohort. However, the “LASSO method” is equivalent to internal validation. Yet, our study should be considered as exploratory, and larger cohorts of patients are needed to confirm our results. In order to allow a more precise evaluation of tumor heterogeneity and to improve reproducibility, CT TA should be performed on the whole tumor instead of its largest cross-sectional area. A fourth limitation is that our study was restricted to patients receiving R-CHOP, so that our results should be further validated for patients with FL treated with obinutuzumab or bendamustine [40]. Finally, TA is highly dependent on the parameters used for CT image acquisition and the algorithm used for evaluation. However, 18F-FDG PET/CT examinations were performed in only one center, with the same acquisition parameters, thus limiting the risk of variability. Furthermore, the software we used for TA has been widely used in other studies, allowing comparison of our results with those of the literature [24,25,28,30,31,32,33].

## 5. Conclusions

The results of our study suggest that, in patients with high-tumor-burden FL, pretreatment CT TA-derived skewness at a fine texture scale and kurtosis without filtration obtained from 18F-FDG PET/CT may act as predictive biomarkers of progression-free survival, notably PFS 24 and TTNT. However, due to the limited number of patients, our results need to be validated through further prospective studies.

## Figures and Tables

**Table 1:** Characteristics of 72 patients.

Variable	Values
Age (years)	61 (24–85; 52–68)
Age > 60 years	38 (52)
Male sex	43 (60)
Histologic grade	
1–2	54 (90)
3a	6 (10)
ECOG > 1	5 (7)
Clinical symptoms	14 (19)
LDH > upper limit of normal	26 (36)
B2 microglobulin > 3 mg/L	26 (38)
Hemoglobin < 12 g/dL	10 (15)
Platelets < 150 × 10^9^/L	11 (17)
Albumin < 40 g/L	28 (51)
Stage III–IV	65 (89)
Nodal sites involvement > 4	46 (63)
Bone marrow involvement	25 (58)
Extranodal sites involvement (other than bone marrow)	38 (53)
LoDLIN > 6 cm	35 (49)
Effusion syndrome	5 (7)
Compression syndrome	13 (18)
Circulating malignant cells	8 (11)
Treatment	
R-CHOP	66 (92)
R-CVP	2 (3)
R-bendamustine	4 (5)
Rituximab maintenance	68 (94)
T-MTV (cm^3)^	381 (12–3329; 155–807)
T-MTV > 510 cm^3^	29 (40)
SUVmax	10.5 (2.7–22.2; 6.1–14.1)
Fails to achieve PFS 24	13 (18)
Death	10 (14)

Quantitative variables are expressed as medians; numbers into parentheses are ranges followed by Q1–Q3. Categorical variables are expressed as raw numbers; numbers in parentheses are percentages. ECOG: Eastern Cooperative Oncology Group; LDH: lactate dehydrogenase; LoDLIN: longest diameter of the largest involved node; R-CHOP: rituximab-cyclophosphamide-doxorubicine-oncovin-prednisone; R-CVP: rituximab-cyclophosphamide-oncovin-prednisone; T-MTV: total metabolic tumor volume; SUVmax: maximum standardized uptake value; PFS24: progression-free-survival at 24 months.

**Table 2:** Multivariable Cox-proportional hazards regression analyses of FL texture parameters and clinical parameters selected via LASSO-penalized Cox regression analysis for predicting progression-free survival.

Parameters	HR (95% CI)	*p* Value
Patient sex	4.81 (1.84, 12.55)	0.001 *
ECOG > 1	5. 5.53 (1.55, 19.79)	0.008 *
FLIPI RC	1.73 (0.95, 3.14)	0.071
TMTV > 510 cm^3^	1.41 (0.64, 3.15)	0.395
Kurtosis_SSF0	1.22 (1.04, 1.44)	0.013 *
Skewness_SSF2	3.72 (1.15, 12.11)	0.028 *

* Indicates a significant difference. ECOG: Eastern Cooperative Oncology Group; FLIPI RC: Follicular Lymphoma International Prognostic Index Risk Category; HR: Hazard Ratio; T-MTV: Total Metabolic Tumor Volume; SSF: Spatial Scale Filter.

**Table 3:** Multivariable Cox-proportional hazards regression analyses of FL texture parameters and clinical parameters selected via LASSO penalized Cox regression analysis for predicting progression-free survival within 24 months.

Parameters	HR (95% CI)	*p* Value
ECOG > 1	3.31 (0.88, 12.33)	0.075
FLIPI RC	2.28 (1.29, 6.11)	0.101
Skewness_SSF2	13.38 (1.29, 138.13)	0.029 *

* Indicates a significant difference. ECOG: Eastern Cooperative Oncology Group; FLIPI RC: Follicular Lymphoma International Prognostic Index Risk Category; SSF: Spatial Scale Filter.

**Table 4:** Multivariable Cox-proportional hazards regression analyses of FL texture parameters and clinical parameters selected via LASSO-penalized Cox regression analysis for predicting time-to-next treatment.

Parameters	HR (95% CI)	*p* Value
Patient sex	3.72 (1.45, 9.53)	0.006 *
ECOG > 1	5.90 (1.58, 21.98)	0.008 *
TMTV > 510 cm^3^	2.07 (0.88, 4.87)	0.093
Kurtosis_SSF0	1.23 (1.04, 1.46)	0.013 *
Skewness_SSF2	5.11 (1.18, 22.13)	0.029 *

* Indicates a significant difference. ECOG: Eastern Cooperative Oncology Group; TMTV: Total Metabolic Tumor Volume; SSF: Spatial Scale Filter.

**Table 5:** Multivariable Cox-proportional hazards regression analyses of FL texture parameters and clinical parameters selected via LASSO-penalized Cox regression analysis for predicting overall survival.

Parameters	HR (95% CI)	*p* Value
Patient sex	19 (1.66, 217.82)	0.018 *
ECOG > 1	3.47 (0.61, 19.86)	0.162
B2-microglobuline	0.87 (0.18, 4.25)	0.868
FLIPI RC	6.43 (1.35, 30.66)	0.019 *
Mean_SSF2	0.92 (0.83, 1.01)	0.097

* Indicates a significant difference. ECOG: Eastern Cooperative Oncology Group; FLIPI RC: Follicular Lymphoma International Prognostic Index Risk Category; SSF: Spatial Scale Filter.

## Data Availability

The data that support the findings of this study are available from the corresponding author [P.S.] upon reasonable request.
